# Population-Level Trends in Emergency Department Encounters for Sexual Assault Preceding and During the COVID-19 Pandemic Across Ontario, Canada

**DOI:** 10.1001/jamanetworkopen.2022.48972

**Published:** 2022-12-29

**Authors:** Katherine A. Muldoon, Robert Talarico, Deshayne B. Fell, Heidi Illingworth, Kari Sampsel, Douglas G. Manuel

**Affiliations:** 1 Ottawa Hospital Research Institute, Clinical Epidemiology Program, Ottawa, Ontario, Canada; 2 ICES, University of Ottawa, Ottawa, Ontario, Canada; 3 Department of Obstetrics and Gynecology, Faculty of Medicine, University of Ottawa, Ottawa, Ontario, Canada; 4 Children’s Hospital of Eastern Ontario Research Institute, Ottawa, Ontario, Canada; 5 School of Epidemiology and Public Health, University of Ottawa, Ottawa, Ontario, Canada; 6 Ottawa Victim Services, Ottawa, Ontario, Canada; 7 Department of Emergency Medicine, Faculty of Medicine, The Ottawa Hospital and University of Ottawa, Ottawa, Ontario, Canada

## Abstract

**Importance:**

Lockdown measures and the stress of the COVID-19 pandemic are factors associated with increased risk of violence, yet there is limited information on trends in emergency department (ED) encounters for sexual assault.

**Objective:**

To compare changes in ED encounters for sexual assault during the COVID-19 pandemic vs prepandemic estimates.

**Design, Setting, and Participants:**

This retrospective, population-based cohort study used linked health administrative data from 197 EDs across Ontario, Canada, representing more than 15 million residents. Participants included all patients who presented to an ED in Ontario from January 11, 2019, to September 10, 2021. Male and female individuals of all ages were included. Data analysis was performed from March to October 2022.

**Exposures:**

Sexual assault, defined through 27 *International Statistical Classification of Diseases and Related Health Problems, Tenth Revision*, procedure and diagnoses codes.

**Main Outcomes and Measures:**

Ten bimonthly time periods were used to compare differences in the frequency and rates of ED encounters for sexual assault between 2020 to 2021 (during the pandemic) compared with baseline prepandemic rates in 2019. Rate differences (RDs) and age adjusted rate ratios (aRRs) and Wald 95% CIs were calculated using Poisson regression.

**Results:**

From January 11, 2019, to September 10, 2021, there were 14 476 656 ED encounters, including 10 523 for sexual assault (9304 [88.4%] among female individuals). The median (IQR) age was 23 (17-33) years for female individuals and 15 (4-29) years for male individuals. Two months before the pandemic, ED encounters increased for sexual assault among female individuals (8.4 vs 6.9 cases per 100 000; RD, 1.51 [95% CI, 1.06 to 1.96]; aRR, 1.22 [95% CI, 1.09 to 1.38]) and male individuals (1.2 vs 1.0 cases per 100 000; RD, 0.19 [95% CI, 0.05 to 0.36]; aRR, 1.19 [95% CI, 0.87 to 1.64]). During the first 2 months of the pandemic, the rates decreased for female individuals (4.2 vs 8.3 cases per 100 000; RD, −4.07 [95% CI, −4.48 to −3.67]; aRR, 0.51 [95% CI, 0.44 to 0.58]) and male individuals (0.5 vs 1.2 cases per 100 000; RD, −0.72 [95% CI, −0.86 to −0.57]; aRR, 0.39 [95% CI, 0.26 to 0.58]). For the remainder of the study period, the rates of sexual assault oscillated, returning to prepandemic levels during the summer months and between COVID-19 waves.

**Conclusions and Relevance:**

These findings suggest that lockdown protocols should evaluate the impact of limited care for sexual assault. Survivors should still present to EDs, especially when clinical care or legal interventions are needed.

## Key Points

**Question:**

What were the population-level changes in emergency department (ED) encounters for sexual assault through 4 waves of the COVID-19 pandemic in 2020 to 2021 compared with 2019 in Ontario, Canada?

**Findings:**

This cohort study of 14 476 656 ED encounters found that ED encounters for sexual assault increased leading up to the state of emergency, decreased immediately after implementation of the lockdown, and then oscillated as the pandemic continued. The pattern was seen across sex, age, income, and community size.

**Meaning:**

These findings suggest that the COVID-19 pandemic may be limiting access to ED care for sexual assault, and survivors should be encouraged to present to EDs when clinical care or legal interventions are needed.

## Teaser

This cohort study compared changes in emergency department (ED) encounters for sexual assault during the COVID-19 pandemic with prepandemic estimates in Ontario, Canada.

## Introduction

The COVID-19 pandemic has changed everyday life and increased many factors associated with the risk of gender-based violence and sexual assault.^1,2,3,4,5^ Key factors include financial stress from job loss or reduced hours, family stress during school and daycare closures, increased elder care, and generalized fear of COVID-19 infection.^6^ Studies show that the majority of cases of sexual assaults occur within intimate partnerships or from known perpetrators,^7^ and the COVID-19 physical distancing protocols pose the unintended consequence of isolating someone with a potentially abusive partner. In addition, stay-at-home orders reduce access to both formal services (eg, health care and legal support) and informal support from family or friends.

There is mixed evidence in the literature describing how the COVID-19 pandemic affected emergency department (ED) encounters for gender-based violence in general, and there are few studies on sexual assault specifically. Medical record reviews^8,9^ from orthopedic trauma and radiology clinics found a decrease in all-cause assault cases, but no difference in physical injuries where the perpetrator was an identified intimate partner. Some studies^10,11^ of ED encounters for assault during COVID-19 reported small decreases in all-cause assault, but did not include specific information on sexual assault. A study^12^ of trends in assault-related penetrating injuries found that ED encounters for gun violence increased during COVID-19 compared with previous years. We conducted a study^7^ on a specialized ED-based sexual assault program and found that the patient volume decreased by half during the first 2 months of the pandemic but the characteristics of the presenting patients were the same (eg, same percentage of female and male patients, cases with police involvement, or intimate partners as perpetrators). With recurring waves of COVID-19, there is concern the pandemic may prevent survivors of sexual assault from seeking medical care and legal interventions through EDs.

In Ontario, Canada, the COVID-19 pandemic state of emergency was declared in March 2020, when stay-at-home directives went into effect. The specific objective of this study was to compare rates of Ontario ED encounters for sexual assault during the pandemic vs the same period preceding the pandemic.

## Methods

### Study Design, Setting, and Population

This population-based cohort study used routinely collected data from ICES, an independent, nonprofit research institute that holds health-related data collected across the province of Ontario, Canada. Ontario represents almost 40% of the Canadian population, with more than 15 million residents.^13^ ICES is authorized to collect and use data for health system analyses, evaluation, and decision support. The study population included male and female individuals of any age living in Ontario with a valid health card. Individuals with implausible ages (>105 years) were excluded. Two timelines are used: (1) a 5-year timeline (January 11, 2016, to September 10, 2021) to display annual patterns in ED encounters for sexual assault, with the COVID-19 pandemic beginning the week of March 11, 2020; and (2) a truncated timeline starting on January 11, 2019, until September 10, 2021, specific to before and during COVID-19 pandemic.

The use of data for this project was authorized under section 45 of Ontario’s Personal Health Information Protection Act, which does not require review by a research ethics board or informed consent. All methods follow the Reporting of Studies Conducted Using Observational Routinely-Collected Data (RECORD) Statement, an extension of the Strengthening the Reporting of Observational Studies in Epidemiology (STROBE) reporting guidelines for cohort studies.

### Data Sources

Data originated from 197 EDs across Ontario. Four databases with information on the entire Ontario population were linked and used for this analysis: (1) National Ambulatory Care Reporting System, which contains information on emergency and ambulatory care encounters; (2) Registered Persons Database, which contains information on patient demographic information, including sex, birth date, death date, and postal code; (3) 2016 Canadian Census with information on neighborhood-level demographic characteristics linked to the patients’ postal code via the Postal Code Conversion File (release date June 2017) that converts 6-digit postal codes into census tracts (approximately 4000 dwellings)^14^; and (4) the COVID-19 Integrated Testing Data set, a comprehensive data set of all available COVID-19 diagnostic laboratory results in Ontario. Laboratory results come from 3 sources in Ontario, including the Ontario Laboratories Information System, distributed testing data from laboratories within the COVID-19 Diagnostic Network compiled by Public Health Ontario, and the Public Health Care and Contact Management Solution (formerly known as the Integrated Public Health Information System). The analyses used deterministic linkage via unique Ontario health care numbers. All direct personal identifiers were removed before the analyses.

### Outcome Measures

The outcome measure was any Ontario ED encounter for sexual assault. The case definition for sexual assault includes 27 *International Statistical Classification of Diseases and Related Health Problems, Tenth Revision *(*ICD-10*) codes that include diagnostic codes, external cause of injury code, and procedural codes, and 18 *ICD-10* codes for suspected child sexual abuse (eTable in Supplement 1).^15^ The codes for suspected childhood sexual abuse included diagnoses of sexually transmitted infections and genital injuries in children younger than 10 years and have been previously published.^16^

### COVID-19 Infections and Waves

The daily rates of COVID-19 infections per 100 000 general population were used to display trends in the COVID-19 pandemic and were collected using standard polymerase chain reaction tests. The COVID-19 waves are displayed and interpreted visually. There are no official dates or discrete categories that confirm the beginning and end of each wave.

### Exposure

**Figure 1.**
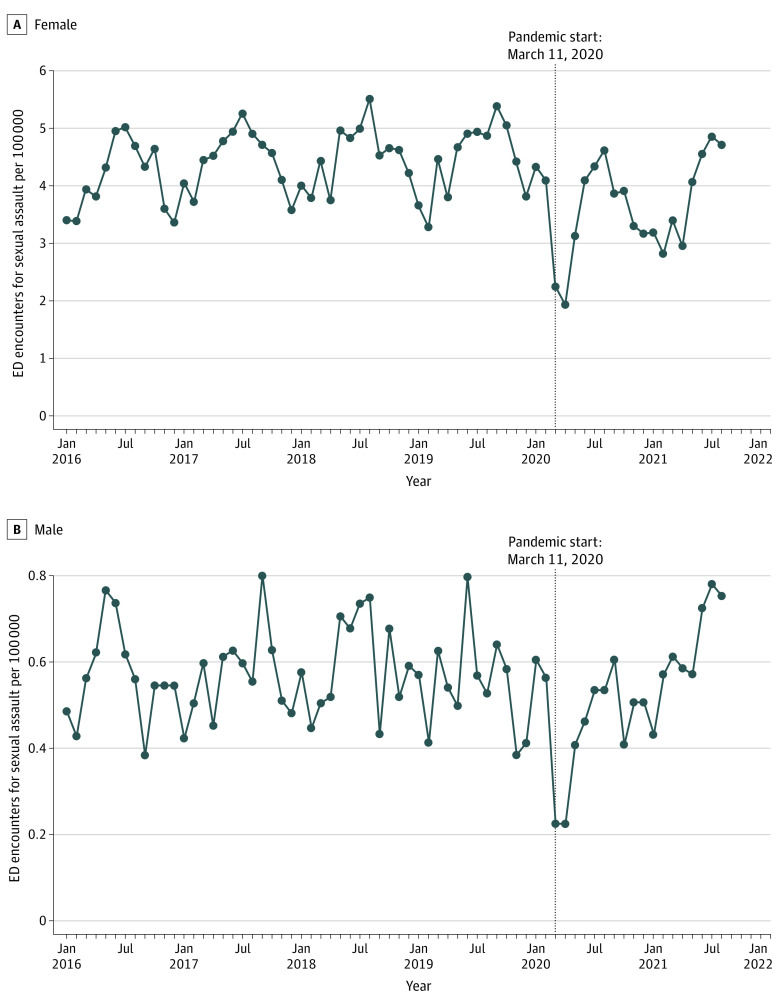
Time Trends of Emergency Department (ED) Encounters for Sexual Assault Between January 11, 2016, and September 10, 2021 The COVID-19 pandemic began the week of March 11, 2020.

The exposure of interest was the COVID-19 pandemic beginning the week of March 11, 2020. Ten bimonthly time periods were used to compare differences in the frequency and rates of ED encounters for sexual assault between 2020 and 2021 compared with 2019. Bimonthly intervals were chosen to avoid small cell sizes. The time trends of ED encounters for sexual assault from January 11, 2016, and September 10, 2021, are displayed in Figure 1. This shows that 2019 is comparable to previous years and is a reliable reference year for the analyses. Time trends for all-cause ED encounters from are available in eFigure 1 in Supplement 1.

### Covariables

Patient-level information included sex (female vs male) and age group in years (0-14, 15-24, 25-35, 35-44, and ≥45 years) and also presented continuously.^17^ Two area-level sociodemographic variables were extracted from the Canadian census: community size, measuring population density ranging from more than 1.5 million to less than 10 000 per census metropolitan area, and neighborhood income quintile, measuring socioeconomic status per census metropolitan area.

### Statistical Analysis

All analyses were conducted using SAS statistical software version 9.4 (SAS Institute)^18^ and were sex stratified. ED encounters for sexual assault are displayed in both frequencies and rates. Daily rates of COVID-19 infection in the general population are displayed against bimonthly ED encounters for sexual assault among male and female patients per 100 000 to descriptive display patterns across the COVID-19 pandemic. The percentage change in ED encounters for sexual assault was calculated by subtracting the 2020 to 2021 bimonthly case counts from 2019 and divided by 2019 and expressed as a percentage to document the change in the volume of cases.

To examine changes in the population rates of ED encounters for sexual assault, rates were calculated per 100 000 general population. Crude rate differences (RDs) and crude rate ratios (RRs) and Wald 95% CIs were calculated using Poisson regression to compare the rate of sexual assault in 2020 to 2021 vs 2019 for each of the 10 time periods. The natural log of the annual denominator was used as the offset term in each model. Age-adjusted Poisson models are presented with adjusted RR (aRR) estimates and 95% CIs. All models were assessed for overdispersion using scaled deviance and Akaike Information Criterion statistic. Data analysis was performed from March to October 2022.

## Results

There were 197 unique EDs in Ontario that submitted data for the analyses. Figure 1 displays the time trends of ED encounters for sexual assault. From January 11, 2016, to September 10, 2021, there were 32 690 216 all-cause ED encounters and 23 128 encounters for sexual assault, including 20 487 sexual assaults (88.6%) among female individuals. There was a seasonal time trend (peaks in the summer and troughs in the winter) and a sharp decrease in March 2020, when the COVID-19 pandemic began. Table 1 shows the general demographic characteristics of ED encounters for sexual assault cases across the 5 years comparing prepandemic with during pandemic. The median (IQR) age was 23 (17-33) years for female individuals and 15 (4-29) years for male individuals.

**Table 1.** Characteristics of Emergency Department Encounters for Sexual Assault Between January 1, 2016, and September 10, 2021

Characteristic	Cases, No. (%)		Total
	Prepandemic, January 11, 2016-March 10, 2020	During pandemic, March 11, 2020-September 10, 2021^a^	
Female cases			
No.	15 672	4815	20 487
Age, median (IQR), y	22 (17-31)	23 (17-33)	22 (17-32)
Age, y			
0-14	2299 (14.7)	751 (15.6)	3050 (14.9)
15-24	7025 (44.8)	1826 (37.9)	8851 (43.2)
25-34	3264 (20.8)	1185 (24.6)	4449 (21.7)
35-44	1770 (11.3)	663 (13.8)	2433 (11.9)
≥45	1314 (8.4)	390 (8.1)	1704 (8.3)
Neighborhood income, quintile			
First (lowest)	5587 (35.6)	1778 (36.9)	7365 (35.9)
Second	3130 (20.0)	1002 (20.8)	4132 (20.2)
Third	2448 (15.6)	740 (15.4)	3188 (15.6)
Fourth	2322 (14.8)	688 (14.3)	3010 (14.7)
Fifth (highest)	1977 (12.6)	555 (11.5)	2532 (12.4)
Missing	208 (1.3)	52 (1.1)	260 (1.3)
Community size			
≥1 500 000	3834 (24.5)	1049 (21.8)	4883 (23.8)
500 000-1 499 999	3147 (20.1)	971 (20.2)	4118 (20.1)
100 000-499 999	4668 (29.8)	1443 (30.0)	6111 (29.8)
10 000-99 999	1667 (10.6)	551 (11.4)	2218 (10.8)
<10 000	2152 (13.7)	753 (15.6)	2905 (14.2)
Missing	204 (1.3)	48 (1.0)	252 (1.2)
Male cases			
No.	1966	675	2641
Age, median (IQR), y	17 (5-28)	15 (4-29)	16 (5-28)
Age, y			
0-14	928 (47.2)	332 (49.2)	1260 (47.7)
15-24	388 (19.7)	106 (15.7)	494 (18.7)
25-34	326 (16.6)	113 (16.7)	439 (16.6)
35-44	159 (8.1)	56 (8.3)	215 (8.1)
≥45	165 (8.4)	68 (10.1)	233 (8.8)
Neighborhood income, quintile			
First (lowest)	632 (32.1)	222 (32.9)	854 (32.3)
Second	402 (20.4)	155 (23.0)	557 (21.1)
Third	336 (17.1)	110 (16.3)	446 (16.9)
Fourth	284 (14.4)	92 (13.6)	376 (14.2)
Fifth (highest)	273 (13.9)	79 (11.7)	352 (13.3)
Missing	39 (2.0)	17 (2.5)	56 (2.1)
Community size			
≥ 1 500 000	592 (30.1)	194 (28.7)	786 (29.8)
500 000-1 499 999	328 (16.7)	119 (17.6)	447 (16.9)
100 000-499 999	558 (28.4)	186 (27.6)	744 (28.2)
10 000-99 999	187 (9.5)	58 (8.6)	245 (9.3)
<10 000	262 (13.3)	101 (15.0)	363 (13.7)
Missing	39 (2.0)	17 (2.5)	56 (2.1)

^a^ The week of March 11, 2020, marks the beginning of the COVID-19 pandemic.

**Figure 2.**
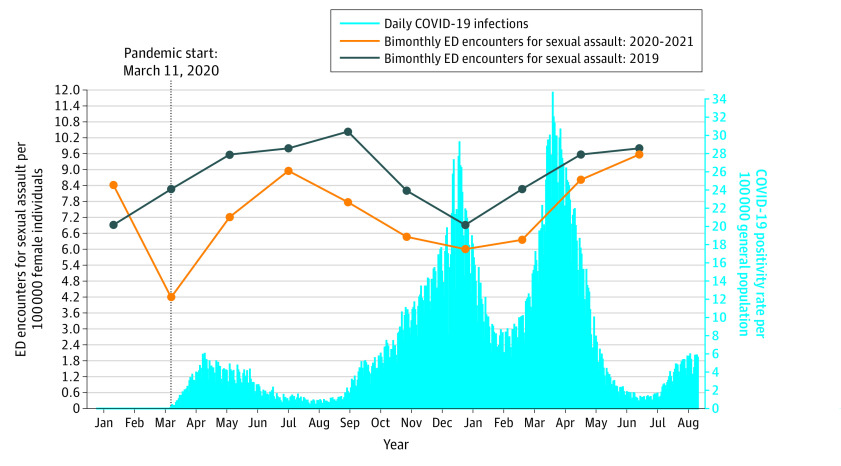
General Population Daily COVID-19 Infection Rates and Monthly Female Sexual Assault Rates per 100 000 in Ontario, Canada, Between January 11, 2019, and September 10, 2021 There were 132 female sexual assault patients with a previous positive diagnosis for COVID-19 prior to admission. ED indicates emergency department.

**Figure 3.**
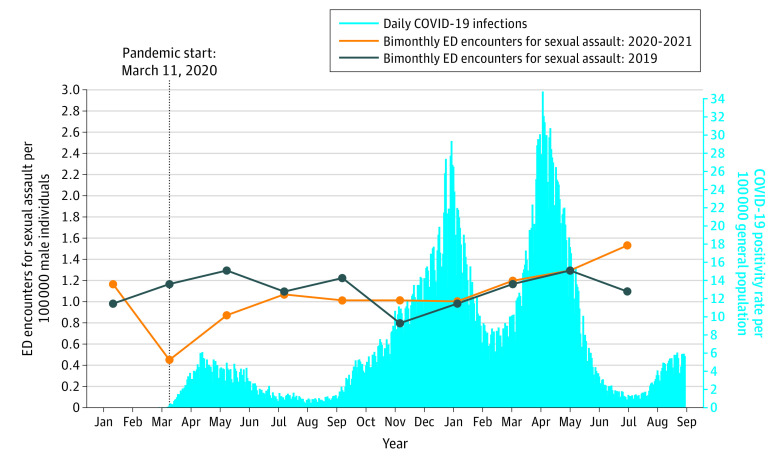
General Population Daily COVID-19 Infection Rates and Monthly Male Sexual Assault Rates per 100 000 in Ontario, Canada, Between January 1, 2019, and September 10, 2021 There were 17 male sexual assault patients with a previous positive diagnosis for COVID-19 prior to admission. ED indicates emergency department.

Between January 11, 2019, and September 10, 2021, there were 14 476 656 ED encounters. Of those, 10 523 were encounters for sexual assault, and 9304 encounters (88.4%) were among female individuals. Figure 2 and Figure 3 display the daily rates of COVID-19 infection in the total population and the bimonthly rates of ED encounters for sexual assault per 100 000, for female and male individuals, respectively. The figures display 4 waves with peaks in April 2020, January 2021, April 2021, and September 2021. There were 132 female and 17 male sexual assault cases with a previous positive diagnosis for COVID-19 before their ED encounter.

Figure 2 displays that the rates of ED encounters for sexual assault among female individuals were above the 2019 estimates in the 2 months preceding the pandemic, then remained below the 2019 estimate for the remainder of the study period. The trends followed the typical seasonal pattern. Figure 3 displays that the rates of ED encounters for sexual assault among male individuals were higher than the 2019 estimates in the 2 months before the pandemic, then returned to similar rates for most of the pandemic, except in the last 2 months when the rates increased.

Table 2 displays the statistical estimates comparing 2020 to 2021 vs 2019. Among female individuals, during the 2 months before the pandemic (January 11 to March 10, 2020), the rates of ED encounters for sexual assault were significantly higher than prepandemic levels (8.4 vs 6.9 cases per 100 000; RD, 1.51 [95% CI, 1.06 to 1.96]; aRR, 1.22 [95% CI, 1.09 to 1.38]). During the first 2 months of the pandemic (March 11 to May 10, 2020) the rates were significantly lower (4.2 vs 8.3 per cases 100 000; RD, −4.07 [95% CI, −4.48 to −3.67]; aRR, 0.51 95% CI, 0.44 to 0.58]), changing by −48.8%. For most of the pandemic, the rates of ED encounters for sexual assault remained significantly lower than prepandemic levels. However, from July 11 to September 10, 2020 (during a trough in the summer, when sexual assaults are generally higher), and May 11 to September 10, 2021 (also during a trough and the summer), the rates returned to prepandemic levels (eFigure 2 in Supplement 1).

**Table 2.** Association Between Emergency Department Encounters for Sexual Assault Comparing COVID-19 Pandemic 2020 to 2021 Time Periods With 2019 Prepandemic

Bimonthly time periods	Year	Events, No.		Change, %	Crude rate per 100 000		Estimates		
		Before pandemic	During pandemic		Before pandemic	During pandemic	Crude RD (95% Wald CI)	Crude RR (95% CI)	Adjusted RR (95% CI)^a^
Female cases									
January 11 to March 10^b^	2020 vs 2019	503	620	23.3	6.9	8.4	1.51 (1.06 to 1.96)	1.22 (1.08 to 1.37)	1.22 (1.09 to 1.38)
March 11 to May 10	2020 vs 2019	601	308	−48.8	8.3	4.2	−4.07 (−4.48 to −3.67)	0.51 (0.44 to 0.58)	0.51 (0.44 to 0.58)
May 11 to July 10	2020 vs 2019	696	531	−23.7	9.6	7.2	−2.35 (−2.82 to −1.88)	0.75 (0.67 to 0.84)	0.76 (0.68 to 0.85)
July 11 to September 10	2020 vs 2019	713	659	−7.6	9.8	9.0	−0.85 (−1.34 to −0.35)	0.91 (0.82 to 1.02)	0.92 (0.82 to 1.02)
September 11 to November 10	2020 vs 2019	759	572	−24.6	10.4	7.8	−2.66 (−3.15 to −2.17)	0.74 (0.67 to 0.83)	0.75 (0.67 to 0.84)
November 11 to January 10	2020-2021 vs 2019	597	476	−20.3	8.2	6.5	−1.74 (−2.18 to −1.30)	0.79 (0.70 to 0.89)	0.79 (0.70 to 0.89)
January 11 to March 10	2021 vs 2019	503	446	−11.3	6.9	6.0	−0.91 (−1.32 to −0.50)	0.87 (0.76 to 0.99)	0.88 (0.77 to 1.00)
March 11 to May 10	2021 vs 2019	601	472	−21.5	8.3	6.4	−1.91 (−2.35 to −1.47)	0.77 (0.68 to 0.87)	0.78 (0.69 to 0.88)
May 11 to July 10	2021 vs 2019	696	640	−8.0	9.6	8.6	−0.96 (−1.44 to −0.47)	0.90 (0.81 to 1.00)	0.91 (0.82 to 1.01)
July 11 to September 10	2021 vs 2019	713	711	−0.3	9.8	9.6	−0.23 (−0.73 to 0.27)	0.98 (0.88 to 1.08)	0.99 (0.89 to 1.09)
Male cases									
January 11 to March 10	2020 vs 2019	69	83	20.3	1.0	1.2	0.19 (0.05 to 0.36)	1.19 (0.86 to 1.64)	1.19 (0.87 to 1.64)
March 11 to May 10	2020 vs 2019	82	32	−61.0	1.2	0.5	−0.72 (−0.86 to −0.57)	0.39 (0.26 to 0.58)	0.39 (0.26 to 0.58)
May 11 to July 10	2020 vs 2019	91	62	−31.9	1.3	0.9	−0.42 (−0.59 to −0.25)	0.67 (0.49 to 0.93)	0.68 (0.49 to 0.93)
July 11 to September 10	2020 vs 2019	77	76	−1.3	1.1	1.1	−0.03 (−0.20 to 0.15)	0.98 (0.71 to 1.34)	0.94 (0.68 to 1.29)
September 11 to November 10	2020 vs 2019	86	72	−16.3	1.2	1.0	−0.21 (−0.39 to −0.04)	0.83 (0.61 to 1.13)	0.87 (0.63 to 1.19)
November 11 to January 10	2020-2021 vs 2019	56	72	28.6	0.8	1.0	0.21 (0.06 to 0.37)	1.27 (0.90 to 1.80)	1.22 (0.86 to 1.72)
January 11 to March 10	2021 vs 2019	69	72	4.4	1.0	1.0	0.02 (−0.14 to 0.18)	1.02 (0.73 to 1.42)	1.03 (0.74 to 1.43)
March 11 to May 10	2021 vs 2019	82	86	4.9	1.2	1.2	0.03 (−0.15 to 0.21)	1.03 (0.73 to 1.42)	1.04 (0.76 to 1.40)
May 11 to July 10	2021 vs 2019	91	93	2.2	1.3	1.3	0.01 (−0.19 to 0.19)	1.00 (0.75 to 1.34)	1.01 (0.76 to 1.35)
July 11 to September 10	2021 vs 2019	77	110	42.9	1.1	1.5	0.43 (0.25 to 0.62)	1.40 (1.05 to 1.87)	1.35 (1.01 to 1.80)

Abbreviations: RD, rate difference; RR, rate ratio.

^a^ Adjusted RR models are adjusted for age.

^b^ January 11 to March 10, 2020, represents the prepandemic period. March 11, 2020, onward is the period during the COVID-19 pandemic.

Among male individuals, during the 2 months before the pandemic (January 11 to March 10, 2020), the rates of ED encounters for sexual assault were higher, but not significantly different, compared with the prepandemic levels (1.2 vs 1.0 cases per 100 000; RD, 0.19 [95% CI, 0.05 to 0.36]; aRR, 1.19 [95% CI, 0.87 to 1.64]). During the first 2 months of the pandemic (March 11 to May 10, 2020) the rates decreased significantly (0.5 vs 1.2 cases per 100 000; RD, −0.72 [95% CI, −0.86 to −0.57]; aRR, 0.39 [95% CI, 0.26 to 0.58]), changing by −61.0%. For 12 months starting July 11, 2020, the rates were the same as in 2019. In the final time period (July 11 to September 10, 2021), the rates were significantly higher than prepandemic levels (1.5 vs 1.1 cases per 100 000; RD, 0.43 [95% CI, 0.25 to 0.62]; aRR, 1.40 [95% CI, 1.05 to 1.87]). Descriptive analyses of the sociodemographic characteristics display a similar pattern where the rates for sexual assault for all age groups, community size, income quintiles were predominantly above prepandemic levels for the 2 months leading up to the pandemic and below expected from the beginning of the pandemic onward (eFigure 3, eFigure 4, and eFigure 5 in Supplement 1).

## Discussion

This cohort study identified a significant increase in rates of ED encounters for sexual assault in the 2 months leading up to the COVID-19 state of emergency and a sharp decrease after lockdown protocols were enacted. This pattern was unexpected and is potentially identifying that violence was increasing in the time shortly before the pandemic but before COVID-19 lockdown protocols limited access to care. This pattern was robust and seen across sex, age group, community size, and income quintile. At several time points, ED encounters for sexual assault among female individuals remained below prepandemic estimates; however, an emerging pattern displayed that rates returned to prepandemic levels during the summer months coinciding with pandemic troughs.^16^

Following the state of emergency, ED encounters for sexual assault decreased for both female (−48.8%) and male (−61.0%) individuals. At the beginning of the pandemic, the population was directly instructed to avoid health care settings, because there was far less information on the transmissibility and severity of COVID-19 infection.^19^ In Ontario, the second and third waves had nearly 5 times the daily rate of COVID-19 infections; however, the public messaging had changed to encourage people to seek care when needed.^20^ The attenuation in rate changes of ED encounters for sexual assault suggests that there were fewer barriers to hospital care in the subsequent waves.

The results of this study support the concern that ongoing waves and restrictions associated with the COVID-19 pandemic may prevent survivors of sexual assault from accessing urgent care. Although some services were able to successfully pivot to remote or virtual care during the pandemic,^21,22,23^ there are clinical services that require in-person medical care and assessment for survivors of sexual assault. Delayed care seeking for sexual assault can negatively impact both the effectiveness of clinical care (eg, emergency contraceptives, prophylaxis and treatment for sexually transmitted infection, or injury assessment) and justice-related processes, including forensic evidence collection and the sexual assault evidence kit.^24,25,26,27^ A decrease in ED encounters for sexual assault can result in long-term clinical, legal, and social consequences that will extend well beyond the urgent phases of the pandemic.^28^

In this study, the first time both male and female ED encounters for sexual assault returned to prepandemic levels was during the summer months in 2020, which also coincided with a trough in COVID-19 cases. This same pattern was also observed in the summer months of 2021. In general, ED encounters for sexual assault follow a seasonal pattern with peaks during the summer months (May to September) and troughs in the winter months (November to January) (Figure 1).^16^ A trough in COVID-19 cases was likely observed in the summer months because the primary mode of COVID-19 transmission is through exposure to respiratory fluids, which is less common when people are outside during the warmer months.^29^ Increases in sexual assault (and other aggravated assaults) are higher during the summer, often associated with more personal contact, longer daylight hours, and heat possibly leading to more aggressive behaviors.^30^ The combination of lifted restrictions and higher risk of sexual assault in the summer highlights the importance of incorporating violence prevention into COVID-19 infection control protocols.

Our study fills an important gap in evidence on ED encounters for sexual assault as the COVID-19 pandemic continues. A comparable study^31^ using National Syndromic Surveillance Program at the US Centers for Disease Control and Prevention investigated trends in ED encounters during COVID-19 and found increases in the rates of self-harm but decreases in encounters for intimate partner violence. Another study^10^ using statewide ED encounters in North Carolina also found a decline in ED encounters for all-cause assault and self-harm, but the study did not differentiate all-cause assault from sexual assault or intimate partner violence. The differing patterns of violence highlight that it is unlikely to find a unifying pattern and supports the need for diverse perspectives and sources of information to characterize how survivors are navigating the COVID-19 pandemic.

Because our unit of analysis is the ED encounter, we do not have information on changes of sexual assault in the general population (which is hypothesized to have increased during the pandemic) and several unknown factors, including changes in the severity of sexual assault cases requiring urgent care, changes in care seeking at nonhospital settings, and changes in timing of care seeking (ie, delayed care seeking).^32^ Research and care patterns on sexual assault are nuanced and can contrast with other forms of violence that require urgent care.^9,12^ For example, ED encounters for life-threatening injuries, such as gunshot wounds, may more accurately reflect the general prevalence of gun violence.^12^ Sexual assaults range in clinical presentation, and many do not result in visible or life-threatening injuries. Information on ED encounters for sexual assault will underestimate the prevalence of sexual assault in the general population. Lack of injuries may also deter survivors from coming to hospitals for care because they may not think it is serious enough for urgent care,^33^ yet the hospital is often the only place where forensic evidence is collected for further legal action.^34^

The increasing risk of violence and sexual assault was anticipated from the beginning of the pandemic in association with financial loss, fear of COVID-19 infection, and social isolation, including being isolated with a potentially controlling and abusive person. A comprehensive response to reducing violence and sexual assault during COVID-19 involves reducing harms among survivors, including improved access to victim services (eg, referrals, accompaniment services, safety planning, and mental health support), clinical care, and social support for leaving dangerous and violent environments.^35^ The primary prevention of sexual assault is more challenging and requires a societal commitment to address a wide range of factors that escalate and sustain violence (and were likely exacerbated during the COVID-19 pandemic).^36,37^ Important commitments include public and social assistance programs that can mitigate factors associated with increased risk of violence (eg, emergency response benefits, financial aid, and subsidized childcare). From the broadest perspective, challenging norms that tolerate violence, fostering open discussion about the common experience of violence, and destigmatizing those exposed to violence remain the most important ways to create safer societies.^38,39^

### Limitations

This study has limitations that should be addressed. Because there were no established dates that measure the waves of the COVID-19 pandemic in Ontario, we can only comment on the patterns of ED encounters for sexual assault throughout the pandemic, and not whether the waves directly contributed to change. In addition, the provincial regulations around lockdown protocols varied across the province and were not consistent enough to create discrete time periods to directly investigate the impacts of lockdowns. It is possible that lockdown dates, rather than COVID-19 infection rates, were more accurate factors associated with changes in ED encounters. Another limitation of this study is the use of data from ambulatory care settings, which represent only the most severe cases of sexual assault requiring urgent care and underestimate the prevalence of sexual assault in the general population. However, our results are generalizable to most hospital-based settings in Canada and likely are generalizable to other regions with similar sociopolitical context. Although we did have area-level information on income, there was no information available on race, ethnicity, or gender diversity, which are all factors that influence exposure to violence.

## Conclusions

This cohort study found that recurring waves of the pandemic may prevent survivors of sexual assault from seeking medical care. There were also seasonal trends identified with increases in ED encounters for sexual assault during the summer months, which coincided with troughs in COVID-19 infections and loosened restrictions. The COVID-19 pandemic has caused many changes to society and health care delivery and access. We recommend that the decision-making regarding the management of the COVID-19 pandemic include antiviolence considerations to evaluate how policies and protocols affect the risk of violence and ensure that those who need health care can access services without concern.

## Notes

Supplement 1. **eTable.*** ICD-10* Codes for Sexual Assault Case Definition **eFigure 1.** Time Trends of All-Cause ED Encounters for Sexual Assault Between January 11, 2016, and September 10, 2021 **eFigure 2.** Percent Change in the Rate of Sexual Assault Cases Stratified by Sex **eFigure 3.** Percent Change in the Rate of Sexual Assault Cases Stratified by Age Group **eFigure 4.** Percent Change in the Rate of Sexual Assault Cases Stratified by Community Size **eFigure 5.** Percent Change in the Rate of Sexual Assault Cases Stratified by Neighborhood Income Quintile

Supplement 2.
Data Sharing Statement
