# Supplementary material for: Population-Level Trends in Emergency Department Encounters for Sexual Assault Preceding and During the COVID-19 Pandemic Across Ontario, Canada
Source: JAMA Netw Open. 2022 Dec 29;5(12):e2248972. doi: 10.1001/jamanetworkopen.2022.48972 (PMC9856789; doi:10.1001/jamanetworkopen.2022.48972)
Supplement: Supplement 1. — eTable. ICD-10 Codes for Sexual Assault Case Definition eFigure 1. Time Trends of All-Cause ED Encounters for Sexual Assault Between January 11, 2016, and September 10, 2021 eFigure 2. Percent Change in the Rate of Sexual Assault Cases Stratified by Sex eFigure 3. Percent Change in the Rate of Sexual Assault Cases Stratified by Age Group eFigure 4. Percent Change in the Rate of Sexual Assault Cases Stratified by Community Size eFigure 5. Percent Change in the Rate of Sexual Assault Cases Stratified by Neighborhood Income Quintile [file jamanetwopen-e2248972-s001.pdf]

Supplemental Online Content

Muldoon KA, Talarico R, Fell DB, Illingworth H, Sampsel K, Manuel DG. Population-level trends in emergency department encounters for sexual assault preceding and during the COVID-19 pandemic across Ontario, Canada. *JAMA Netw Open*. 2022;5(12):e2248972. doi:10.1001/jamanetworkopen.2022.48972

eTable. *ICD-10* Codes for Sexual Assault Case Definition

eFigure 1. Time Trends of All-Cause ED Encounters for Sexual Assault Between January 11, 2016, and September 10, 2021

eFigure 2. Percent Change in the Rate of Sexual Assault Cases Stratified by Sex

eFigure 3. Percent Change in the Rate of Sexual Assault Cases Stratified by Age Group

eFigure 4. Percent Change in the Rate of Sexual Assault Cases Stratified by Community Size

eFigure 5. Percent Change in the Rate of Sexual Assault Cases Stratified by Neighborhood Income Quintile

This supplemental material has been provided by the authors to give readers additional information about their work.

© 2022 Muldoon KA et al. *JAMA Network Open*.

Supplemental material

eTable. *ICD-10* Codes for Sexual Assault Case Definition

Codes	Label	SGBV_3
T742	Sexual abuse	Sexual assault
Y05	Sexual assault by bodily force	Sexual assault
Z044	Examination and observation following alleged rape and seduction	Sexual assault
Z0450	Examination and observation following alleged adult sexual and physical abuse	Sexual assault
Z0451	Examination and observation following alleged child sexual and physical abuse	Sexual assault
Z614	Problems related to alleged sexual abuse of child by person within primary support group	Sexual assault
Z615	Problems related to alleged sexual abuse of child by person outside primary support group	Sexual assault
Z702	Counselling related to sexual behaviour and orientation of third party	Sexual assault
Z703	Counselling related to combined concerns regarding sexual attitude, behaviour and orientation	Sexual assault
A540	Gonococcal infection of lower genitourinary tract without periurethral or accessory gland abs	Suspected sexual abuse in children 10 and under
A541	Gonococcal infection of lower genitourinary tract with periurethral and accessory gland absce	Suspected sexual abuse in children 10 and under
A542	Gonococcal pelviperitonitis and other gonococcal genitourinary infections	Suspected sexual abuse in children 10 and under
A544	Gonococcal infection of musculoskeletal system	Suspected sexual abuse in children 10 and under
A545	Gonococcal pharyngitis	Suspected sexual abuse in children 10 and under
A546	Gonococcal infection of anus and rectum	Suspected sexual abuse in children 10 and under
A548	Other gonococcal infections	Suspected sexual abuse in children 10 and under
A5486	Gonococcal sepsis	Suspected sexual abuse in children 10 and under
A5488	Other gonococcal infections	Suspected sexual abuse in children 10 and under
A549	Gonococcal infection, unspecified	Suspected sexual abuse in children 10 and under
A600	Herpesviral infection of genitalia and urogenital tract	Suspected sexual abuse in children 10 and under
A609	Anogenital herpesviral infection, unspecified	Suspected sexual abuse in children 10 and under
N739	Female pelvic inflammatory disease, unspecified	Suspected sexual abuse in children 10 and under
S3020	Contusion of penis	Suspected sexual abuse in children 10 and under
S3021	Contusion of scrotum and testes	Suspected sexual abuse in children 10 and under
S3022	Contusion of vagina and vulva	Suspected sexual abuse in children 10 and under
S3028	Contusion of other external genital organs	Suspected sexual abuse in children 10 and under
S3029	Contusion of unspecified external genital organs	Suspected sexual abuse in children 10 and under

© 2022 Muldoon KA et al. *JAMA Network Open.*

eFigure 1. Time Trends of All-Cause ED Encounters for Sexual Assault Between January 11, 2016, and September 10, 2021

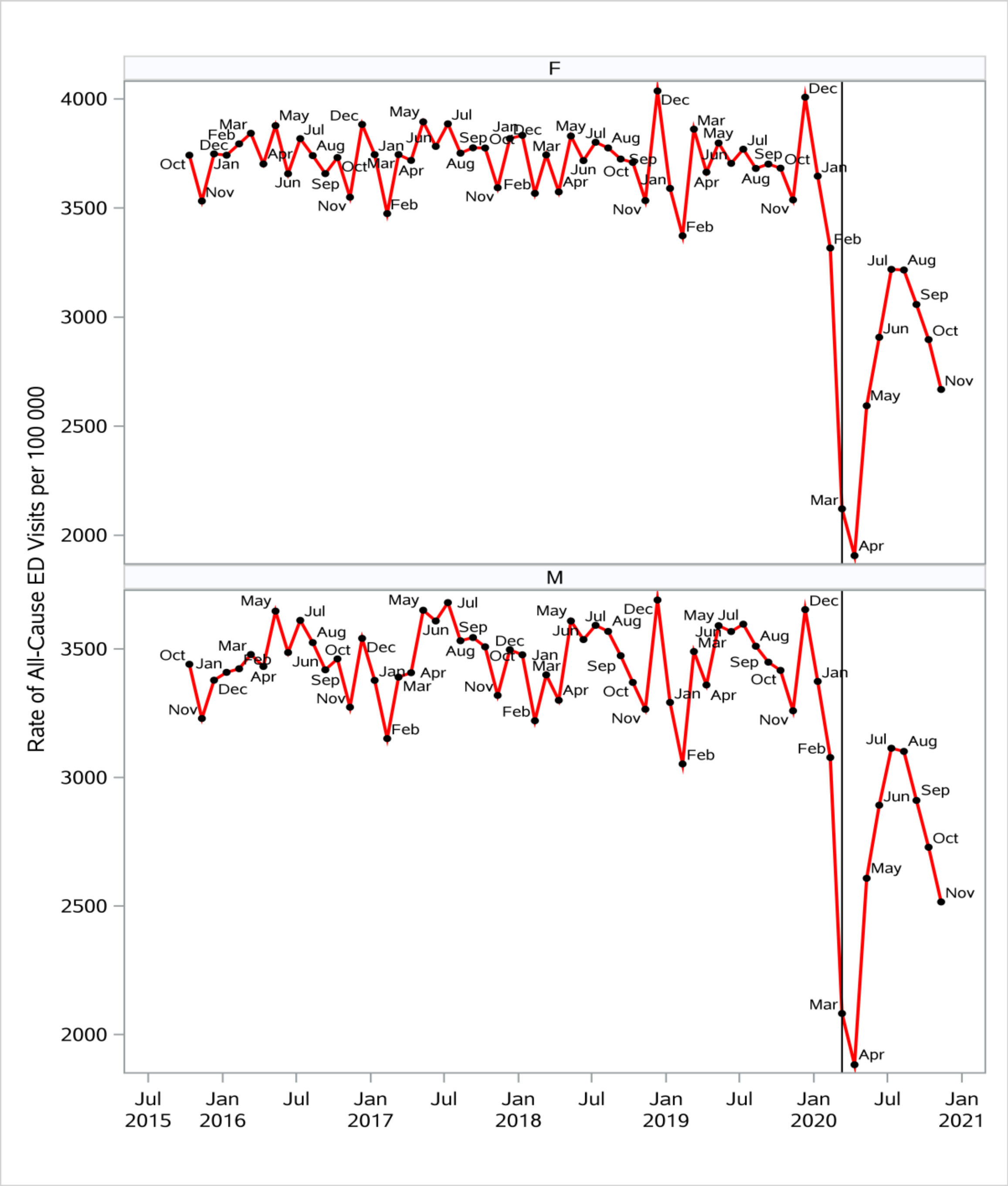

© 2022 Muldoon KA et al. *JAMA Network Open.*

eFigure 2. Percent Change in the Rate of Sexual Assault Cases Stratified by Sex

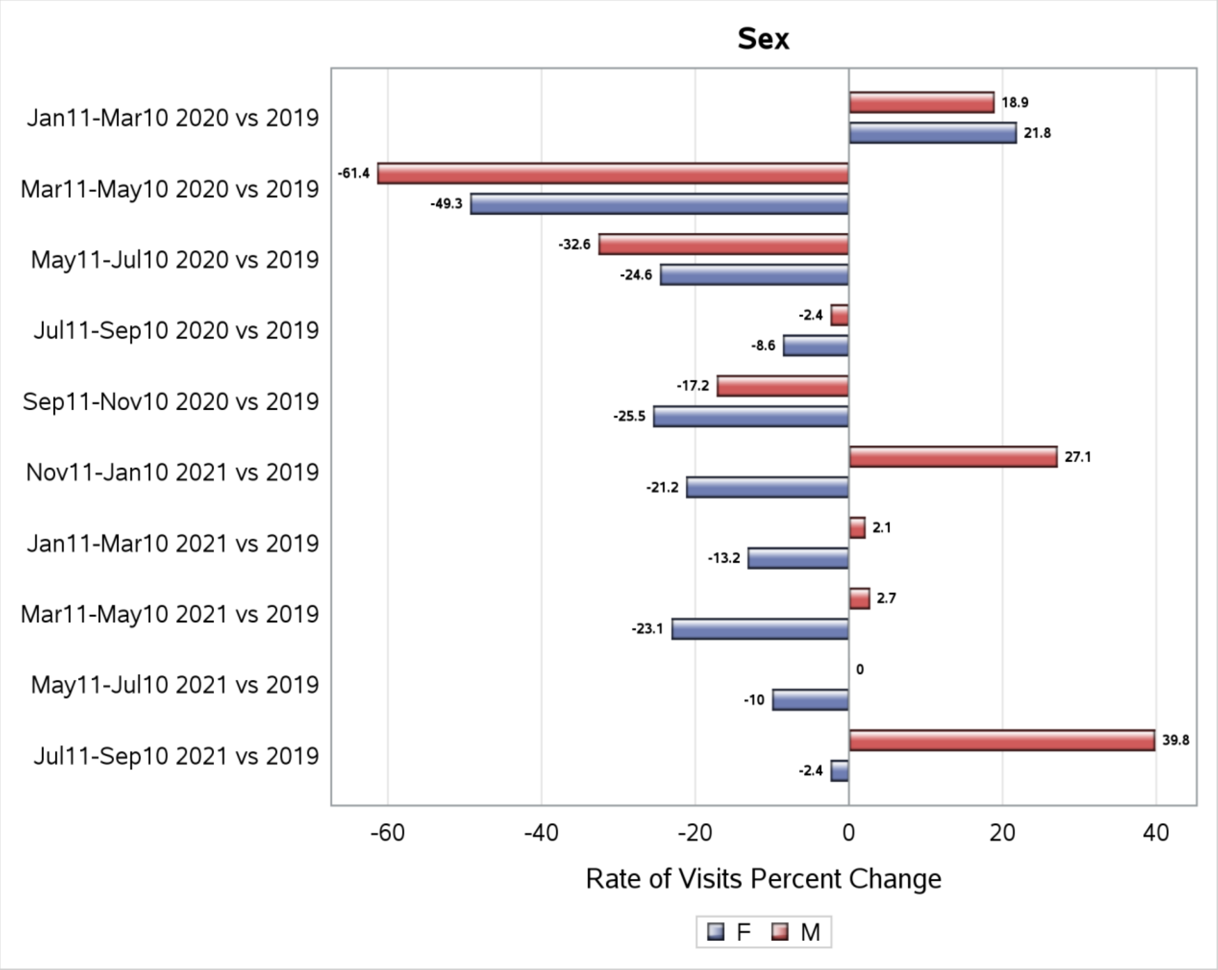

NB: Percent changes are calculated by subtracting the 2020/21 counts from 2019 and dividing by 2019, and expressed as a percentage. The positive percent changes to the right of zero indicate that the 2020/21 are higher than 2019. To the left of zero indicate that the 2020/21 rates are lower than 2019. January 11th to March 10th, 2020, are two months pre-pandemic and the rates are higher than 2019. March 11th 2020 onwards are months during the pandemic and the rates are mostly lower than 2019 for females. Males are lower until September 11th 2020 and then the rates increase particularly in July 11th 2021.

© 2022 Muldoon KA et al. *JAMA Network Open.*

eFigure 3. Percent Change in the Rate of Sexual Assault Cases Stratified by Age Group

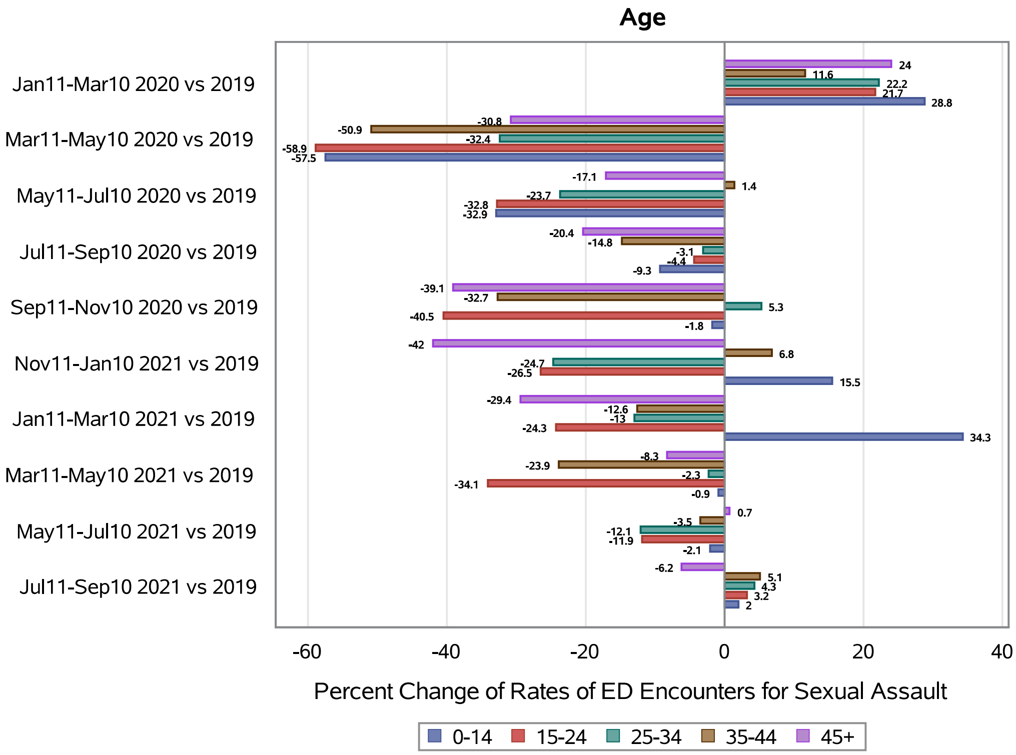

Percent changes are calculated by subtracting the 2020/21 counts from 2019 and dividing by 2019, and expressed as a percentage. The positive percent changes to the right of zero indicate that the 2020/21 are higher than 2019. To the left of zero indicate that the 2020/21 rates are lower than 2019. January 11th to March 10th, 2020, are two months pre-pandemic and the rates are higher than 2019. March 11th 2020 onwards are months during the pandemic and the rates are mostly lower than 2019.

Due to small cell sizes, the age groups have been grouped into 5 categories. Catgories 0-14 years and 45+ years are heterogeneous categories and should be interpreted with caution

© 2022 Muldoon KA et al. *JAMA Network Open.*

eFigure 4. Percent Change in the Rate of Sexual Assault Cases Stratified by Community Size

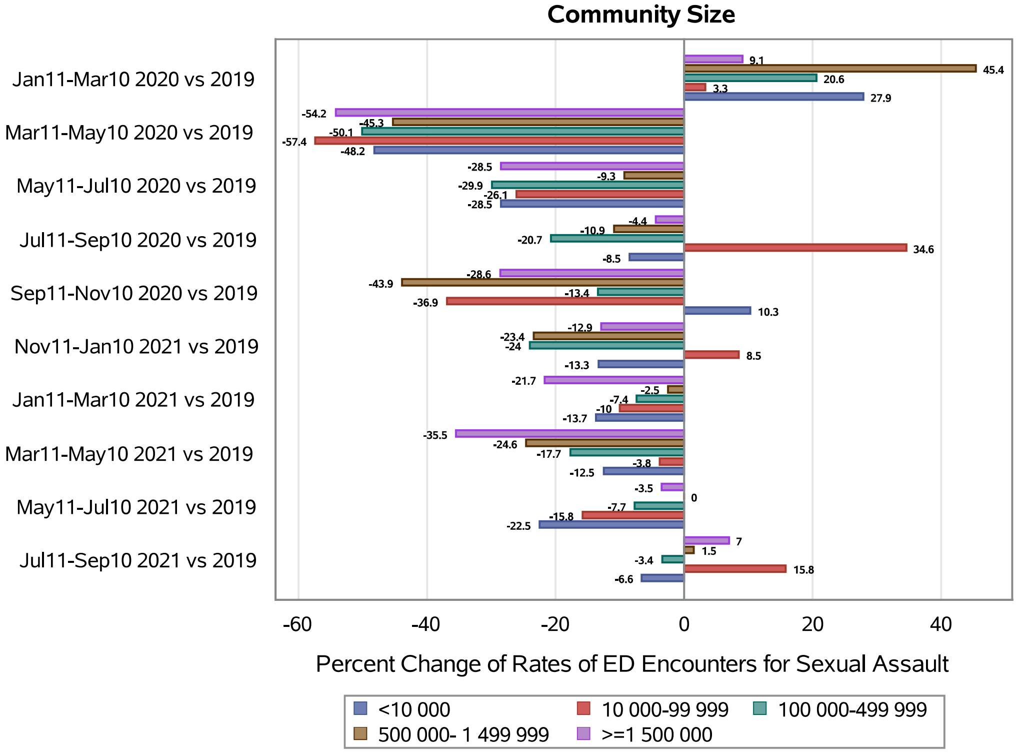

NB: Percent changes are calculated by subtracting the 2020/21 counts from 2019 and dividing by 2019, and expressed as a percentage. The positive percent changes to the right of zero indicate that the 2020/21 are higher than 2019. To the left of zero indicate that the 2020/21 rates are lower than 2019. January 11th to March 10th, 2020, are two months pre-pandemic and the rates are higher than 2019. March 11th 2020 onwards are months during the pandemic and the rates are mostly lower than 2019.

Community size is derived from the Canadian Census and measures population density.

© 2022 Muldoon KA et al. *JAMA Network Open.*

eFigure 5. Percent Change in the Rate of Sexual Assault Cases Stratified by Neighborhood Income Quintile

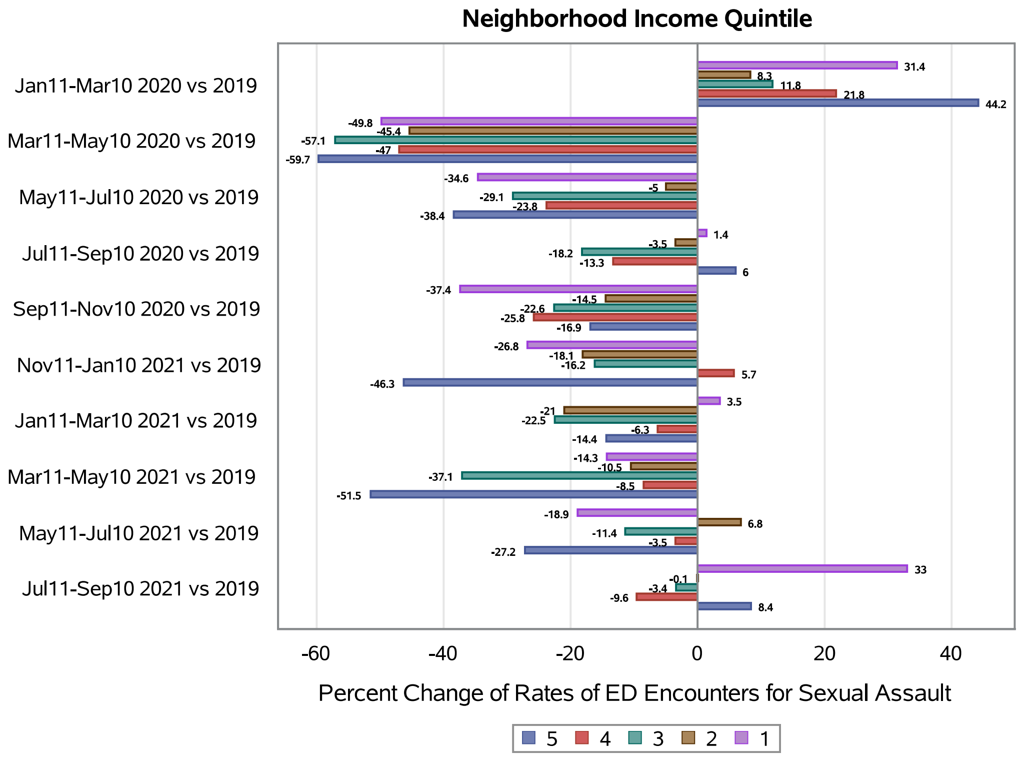

NB: Percent changes are calculated by subtracting the 2020/21 counts from 2019 and dividing by 2019, and expressed as a percentage. The positive percent changes to the right of zero indicate that the 2020/21 are higher than 2019. To the left of zero indicate that the 2020/21 rates are lower than 2019. January 11th to March 10th, 2020, are two months pre-pandemic and the rates are higher than 2019. March 11th 2020 onwards are months during the pandemic and the rates are mostly lower than 2019.

Neighbourhood Income Quintile is derived from the Canadian Census. Category 5 is the highest quintile and 1 is the lowest quintile.

© 2022 Muldoon KA et al. *JAMA Network Open.*
