# Supplementary material for: Population-Level Trends in Emergency Department Encounters for Sexual Assault Preceding and During the COVID-19 Pandemic Across Ontario, Canada
Source: JAMA Netw Open. 2022 Dec 29;5(12):e2248972. doi: 10.1001/jamanetworkopen.2022.48972 (PMC9856789; doi:10.1001/jamanetworkopen.2022.48972)
Supplement: Supplement 2. — Data Sharing Statement [file jamanetwopen-e2248972-s002.pdf]

Data Sharing Statement

Muldoon. Population-Level Trends in Emergency Department Encounters for Sexual Assault Preceding and During the COVID-19 Pandemic Across Ontario, Canada. *JAMA Netw Open*. Published December 29, 2022. doi:10.1001/jamanetworkopen.2022.48972

Data
Data available: No

Additional Information
Explanation for why data not available: The individual data are protected by the Ministry of Health and Longterm Care. The data dictionary and data creation plan are available upon request
